# Supplementary material for: Multiphase detection of crucial biological amines using a 2,4,6-tristyrylpyrylium dye
Source: Commun Chem. 2025 Mar 15;8:81. doi: 10.1038/s42004-025-01459-5 (PMC11910641; doi:10.1038/s42004-025-01459-5)
Supplement: Supplementary file 2 — Supplementary Information [file 42004_2025_1459_MOESM2_ESM.pdf]

Multiphase Detection of Crucial Biological Amines using a 2,4,6-Tristyrylpyrylium Dye

Shivani Tripathi,
Banchhanidhi Prusti, and
Manab Chakravarty*

Department of Chemistry, BITS-Pilani, Hyderabad
Campus, Jawahar Nagar, Shamirpet, Hyderabad-500078,
India

Contents

1. (TGA) and solid-state UV-Vis/Fl spectroscopy of PyTPA---S2
2. Photostability of **PyTPA** in solution---S2
3. Experimental and theoretical absorption study---S3
4. Emission spectra of **PyTPA** (10 µM) with different Amines---S3
5. Absorbance spectrum for selectivity among different class of amines and phenols------------------------S4
6. Absorbance response of the dye upon mixing different amines--S4
7. Emission spectra of PyTPA, upon titration with various amines--S5
8. Limit of detection plot for all amines---S6-S8
9. Paper Strip (PyTPA@WP) characterization--S9
10. Job's plot of **PyTPA**-- S10
11. a) LC-MS analysis of **PyTPA** with variety of amines ---S11
12. b) ^{1}H NMR analysis of PyTPA with Cadaverine--S11
13. XPS details--S12
14. LC-MS spectra of **PyTPA** with Nicotine --S12
15. Excited state Lifetime studies---S13
16. A comparative literature---S14-S15
17. Characterisation of **PyTPA** via HRMS, ^{1}HNMR, ^{13}CNMR and IR ---S16-S17

Thermogravimetric analysis (TGA) and solid-state UV-Vis/Fl spectroscopy of PyTPA

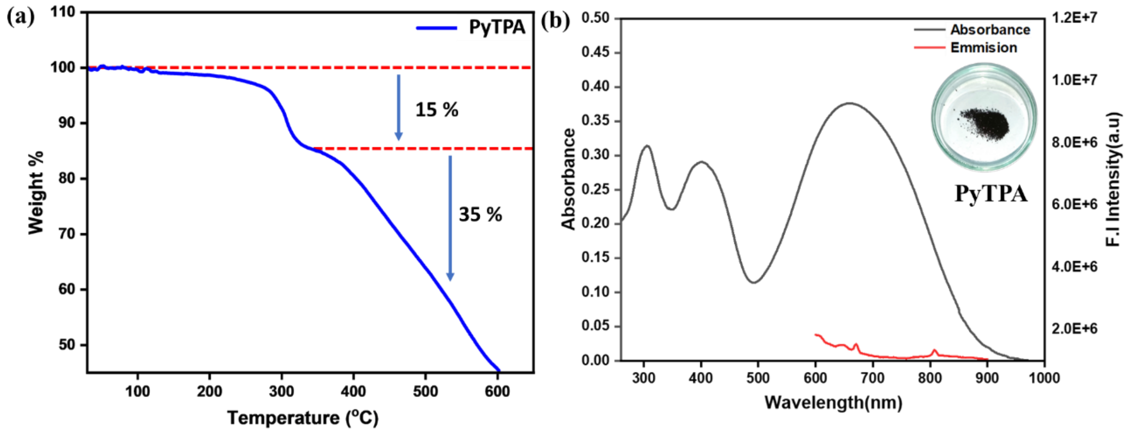

Fig. S1: (a) TGA plot (b) Solid state UV-Vis and emission spectrum for **PyTPA**

Photostability of PyTPA in solution.

Absorbance of **PyTPA** (10 µM) in MeCN was recorded at different intervals of time for 48 hours, and almost no change in absorbance was observed, upon addition of 0.5 µM cadaverine in the same solution of **PyTPA,** absorbance instantaneously dropped down further absorbance was recorded at different time intervals till 72 hours, where absorbance remained constant till 72h. Therefore, the interactions are instantaneous. It indicates the good photostability of **PyTPA**.

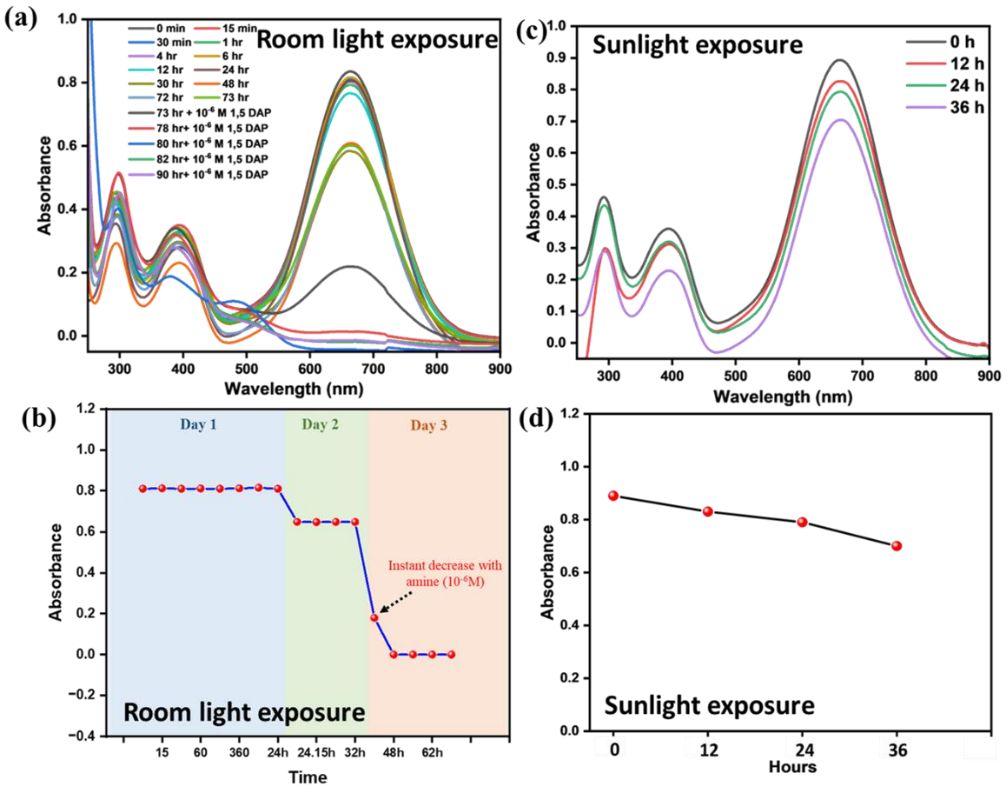

Fig. S2: (a) Absorption spectra of **PyTPA** (10 µM in MeCN) after exposing under room light for 72 h and after the addition of 1,5-Dap (cadaverine, CAD), (b) absorbance vs time plot of **PyTPA** and after the addition of cadaverine (c) Absorbance spectrum of **PyTPA** (10 µM in MeCN) after exposing sunlight for 3 days (400-10,00 lux) (d) absorbance vs time plot of **PyTPA** when exposing sunlight for 3 days.

Experimental and theoretical absorption study

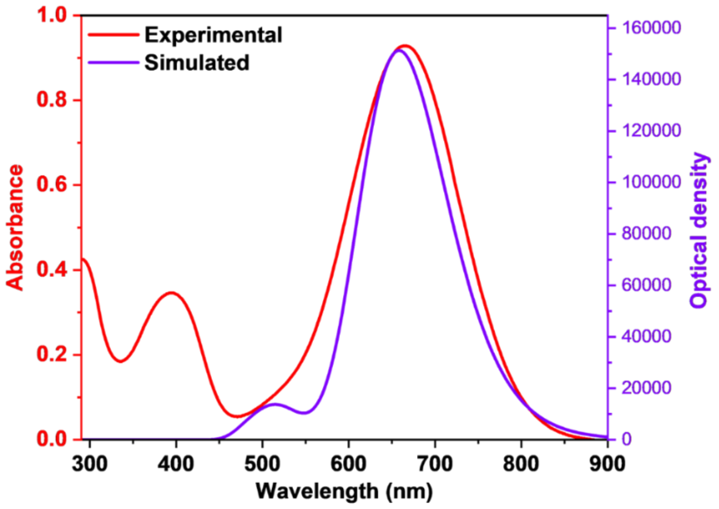

Fig. S3: Experimental and theoretical absorption spectra for **PyTPA** in MeCN.

Table S1: Optical properties comparing experimental and theoretical data.

Probe	λ_{abs} (nm) Experimental	λ_{abs} (nm) Simulated	Oscillation strength (*f*)	Orbital Contribution
**PyTPA**	663	655	0.5470	H→L (92%)<br>H-1→L (3)

The TD-DFT calculations were performed using ωb97xd/6-31+g(d,p)/acetonitrile (CPCM).

Emission spectrum of PyTPA (10 μM) with different amines

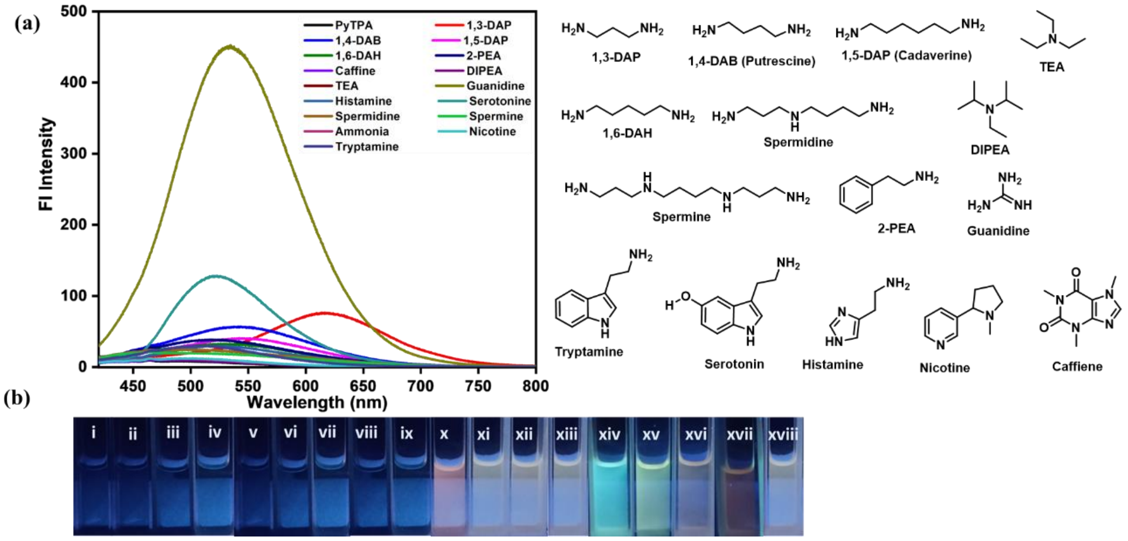

Fig. S4: (a) Emission spectra of **PyTPA** (10 μM) with different Amines (λ_{exi}= 410 nm) (b) photograph of **PyTPA** (10 μM) with different amines under UV (365 nm) where; (i) **PyTPA** (10 μM),(ii) DMAc (iii) caffeine, (iv) DIPEA, (v) nicotine, (vi) ammonia, (vii) triethylamine, (viii) tryptamine, (ix) serotonin (x) 1,6-DAH, (xi) CAD, (xii) spermine, (xiii) spermidine, (xiv) guanidine, (xv) histamine, (xvi) PUT, (xvii) 1,3-DAP, (xviii) 2-PEA.

Absorbance spectrum for checking the selectivity among different class of amines and phenols.

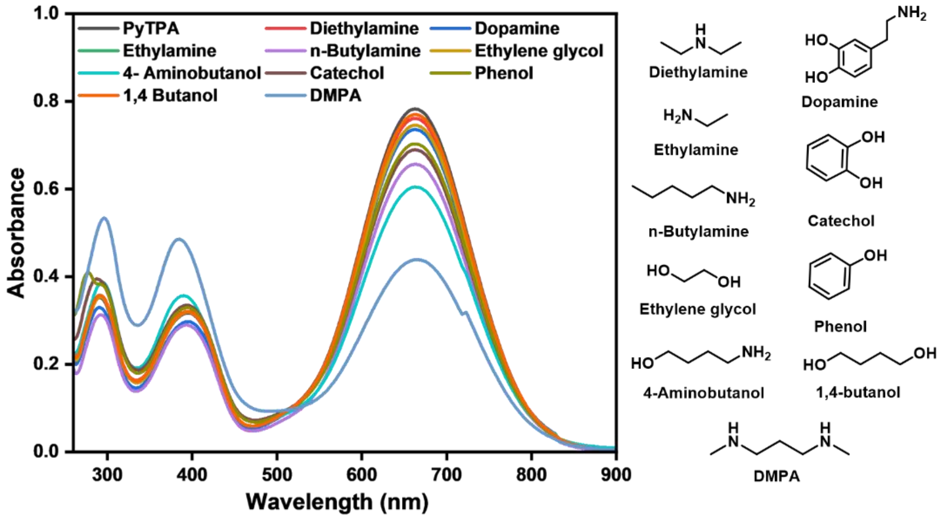

Fig. S5: Absorbance spectrum of PyTPA (10 µM) with different classes of amines and phenols

Absorbance response of the dye upon mixing different amines

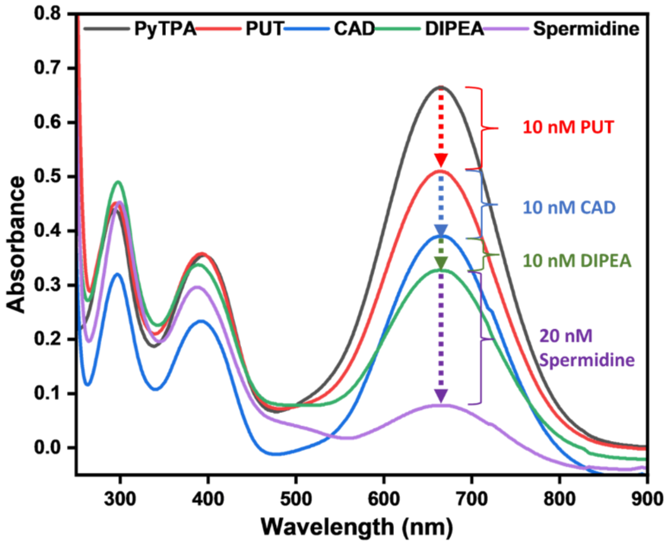

Fig. S6: Absorbance response of the **PyTPA** (**10 µM** in MeCN) in mixture of amines

Emission spectra of PyTPA, upon titration with various amines

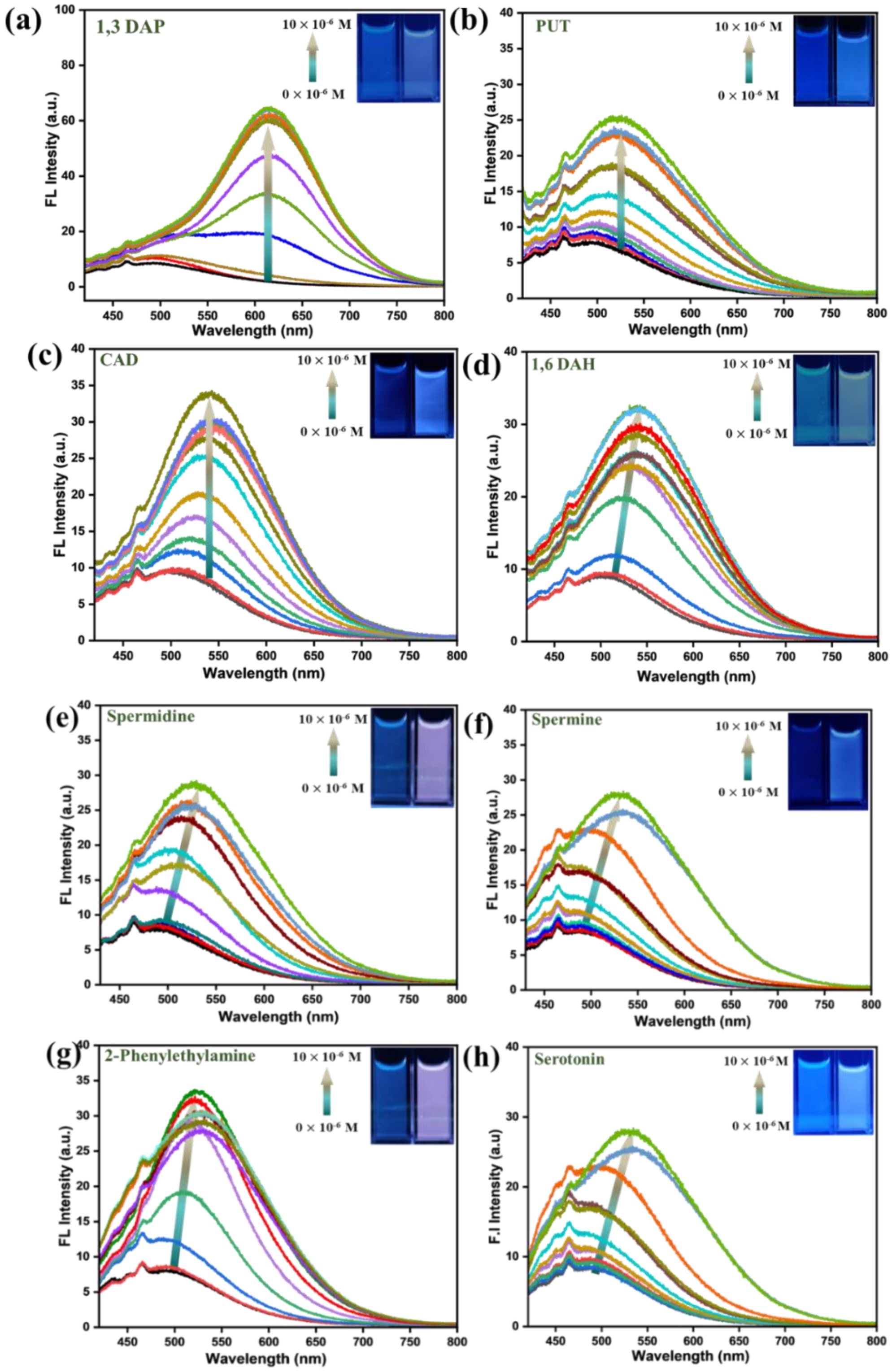
(a)
1,3 DAP
10 × 10⁻⁶ M
0 × 10⁻⁶ M
FL Intesity (a.u.)
Wavelength (nm)
(b)
PUT
FL Intensity (a.u.)
Wavelength (nm)
(c)
CAD
FL Intensity (a.u.)
Wavelength (nm)
(d)
1,6 DAH
FL Intensity (a.u.)
Wavelength (nm)
(e)
Spermidine
FL Intensity (a.u.)
Wavelength (nm)
(f)
Spermine
FL Intensity (a.u.)
Wavelength (nm)
(g)
2-Phenylethylamine
FL Intensity (a.u.)
Wavelength (nm)
(h)
Serotonin
F.I Intensity (a.u)
Wavelength (nm)

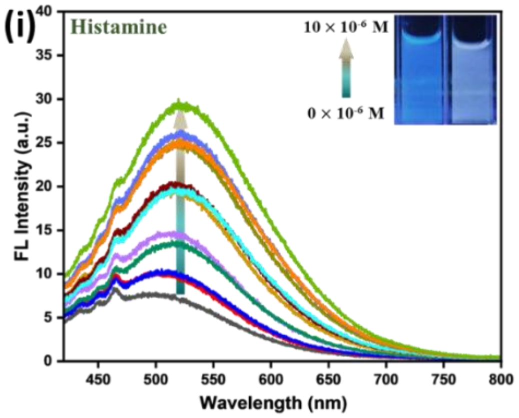

Fig. S7: Emission spectra of **PyTPA**, upon titration with various amines (a) 1,3-DAP, (b) PUT, (c) CAD, (d) 1,6-DAH, (e) spermidine, (f) spermine, (g) 2-PEA, (h) serotonin, and (i) histamine,

Limit of detection plot for all amines.

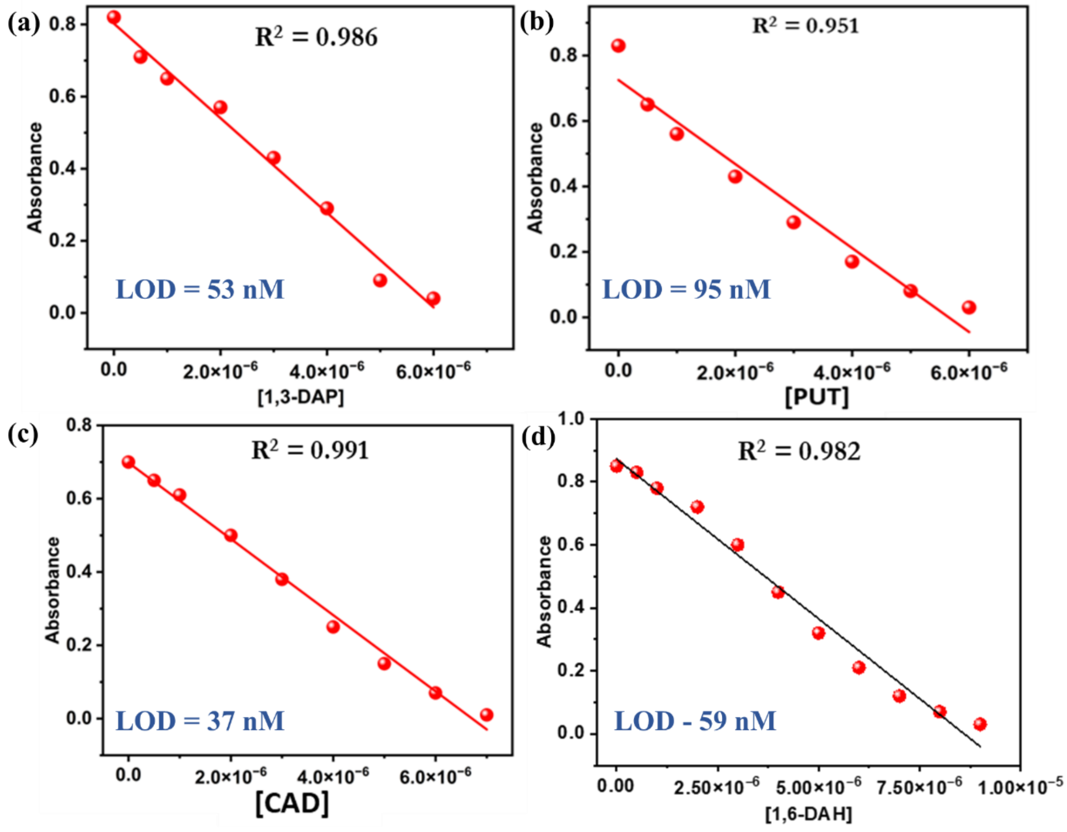

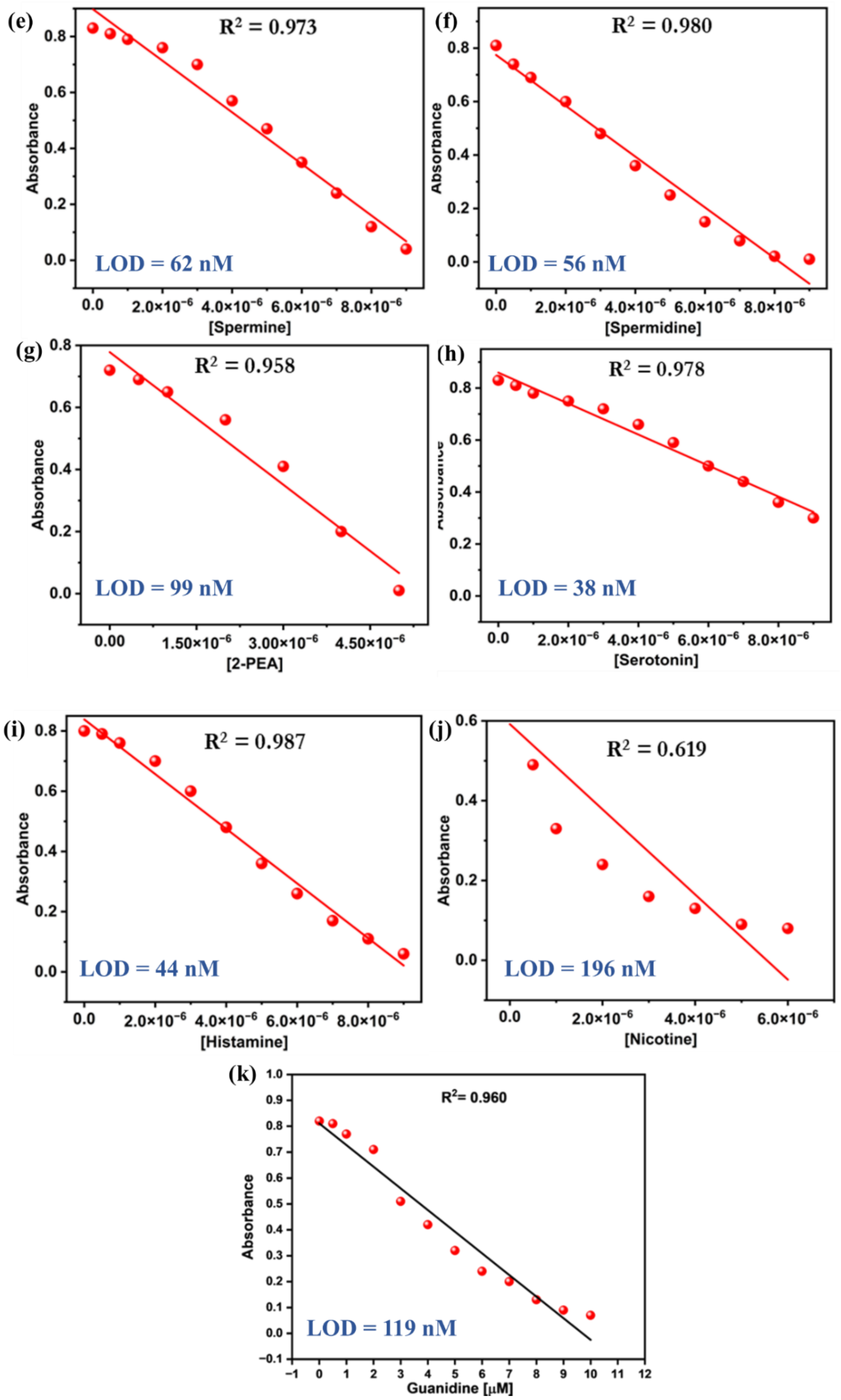

Fig. S8: Limit of detection plot for (a) 1,3-DAP, (b) PUT, (c) CAD, (d) 1,6-DAH, (e) spermine, (f) spermidine, (g) 2-PEA, (h) serotonin, (i) histamine, (j) nicotine and (k) guanidine.

Calculation of the Limit of Detection (LOD):

The standard deviation and slope were obtained by plotting the ratio of absorbance intensity at 640 nm with concentration of amine **LOD = 3.3 σ/K**

Where, σ = Standard error of the linear fitting (highlighted in red in the graph below) and K = Slope

e.g. For **1,3-DAP,** σ = 0.01962 and K = 1.22 x 10^6 M^{-1}, thus LOD= $\frac{3.3\times0.01962}{1.22\times10^6}$ = 53 x 10^{-9} M

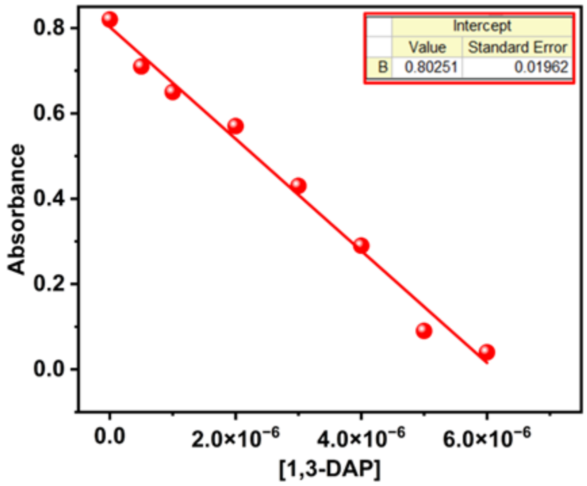

We have titrated in the range of 0.5-10 µM of the analyte to the sensor solution. Thus, the minimum concentration of the calibration was 0.5 µM.

Table S2: LOD parameters for all analytes (solution state)

**S.No.**	**Analyte**	**K (Calibration Slope)**	**σ (Standard error)**	**LOD (nM)**
A	1,3-DAP	1.22 x 10^6	0.01962	53
B	PUT	1.28 x 10^6	0.03679	95
C	CAD	1.18 x 10^6	0.01307	37
D	1,6-DAH	1.23 x 10^6	0.02199	59
E	Spermidine	1.35 x 10^6	0.02288	56
F	Spermine	1.31 x 10^6	0.02457	62
G	2-PEA	1.12 x 10^6	0.03367	99
H	Serotonin	1.21 x 10^6	0.01408	38
I	Histamine	1.22 x 10^6	0.01634	44
J	Nicotine	1.72 x 10^6	0.10236	196
K	Guanidine	0.83 x 10^6	0.03000	119

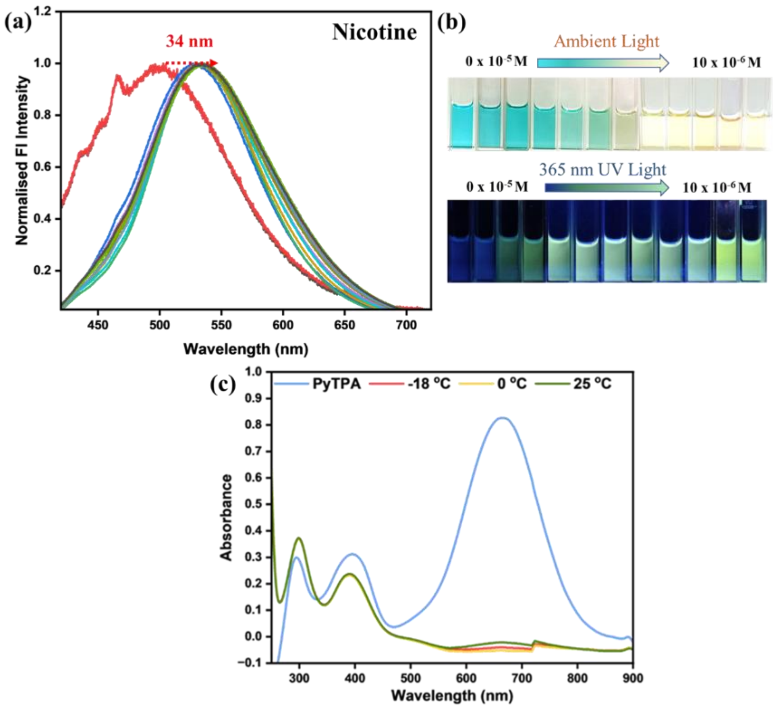

Fig. S9: (a) Normalized FL spectrum upon gradual addition of nicotine, (b) photograph of PyTPA (10 μM) upon gradual addition of nicotine (solution of PyTPA (10 μM in MeCN) with different amine concentration was heated at 40 °C for 2 h and further cooled to room temperature and emission spectra were recorded) (c) Absorbance spectra of the PyTPA (10 μM in MeCN) + CAD (10 μM in DMAc) at three different temperatures.

Paper Strip (PyTPA@WP) characterization

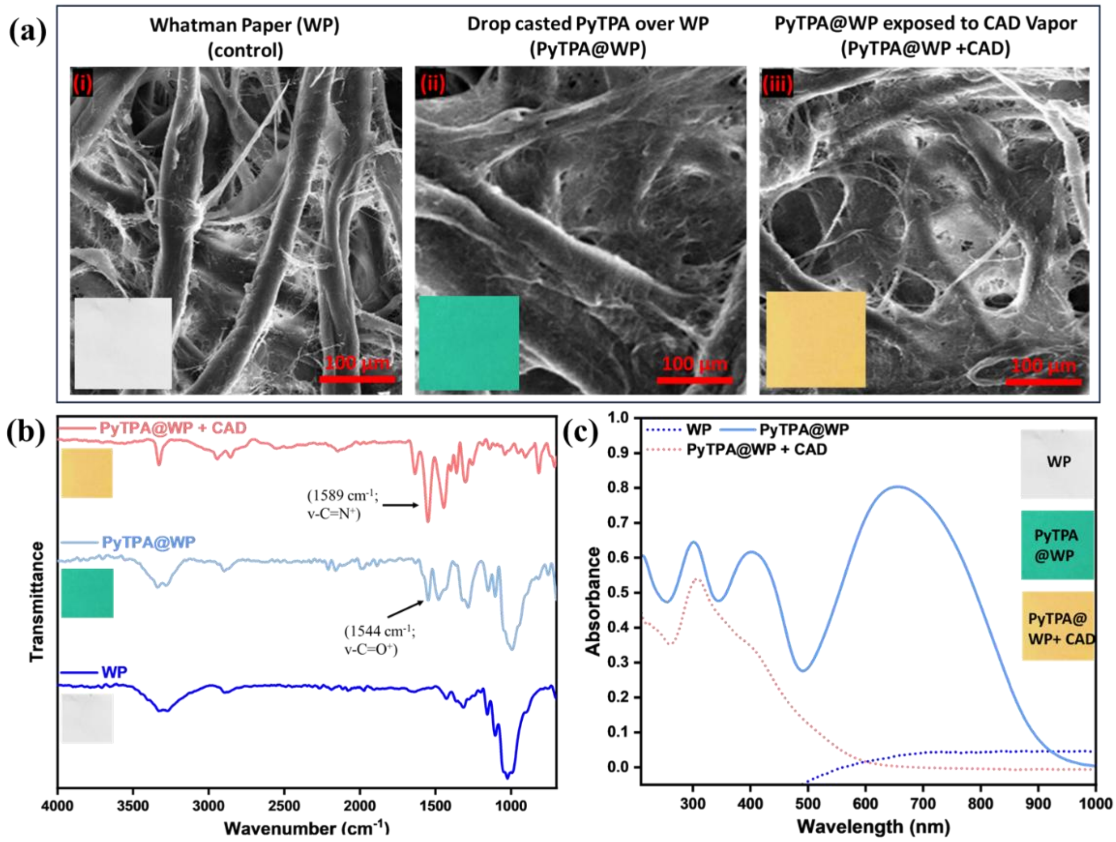

Fig. S10: (a) SEM Image of (i) Whatman filter paper (WP) (ii) PyTPA drop casted WP(PyTPA@WP) (iii) PyTPA Dorp cast paper upon exposure of CAD vapours(PyTPA@WP+CAD).(b) comparative IR s spectra of WP, PyTPA@WP, and PyTPA@WP+CAD (c) comparative UV-Vis spectrum of WP, PyTPA@WP, and PyTPA@WP+CAD.

Job's plot of PyTPA.

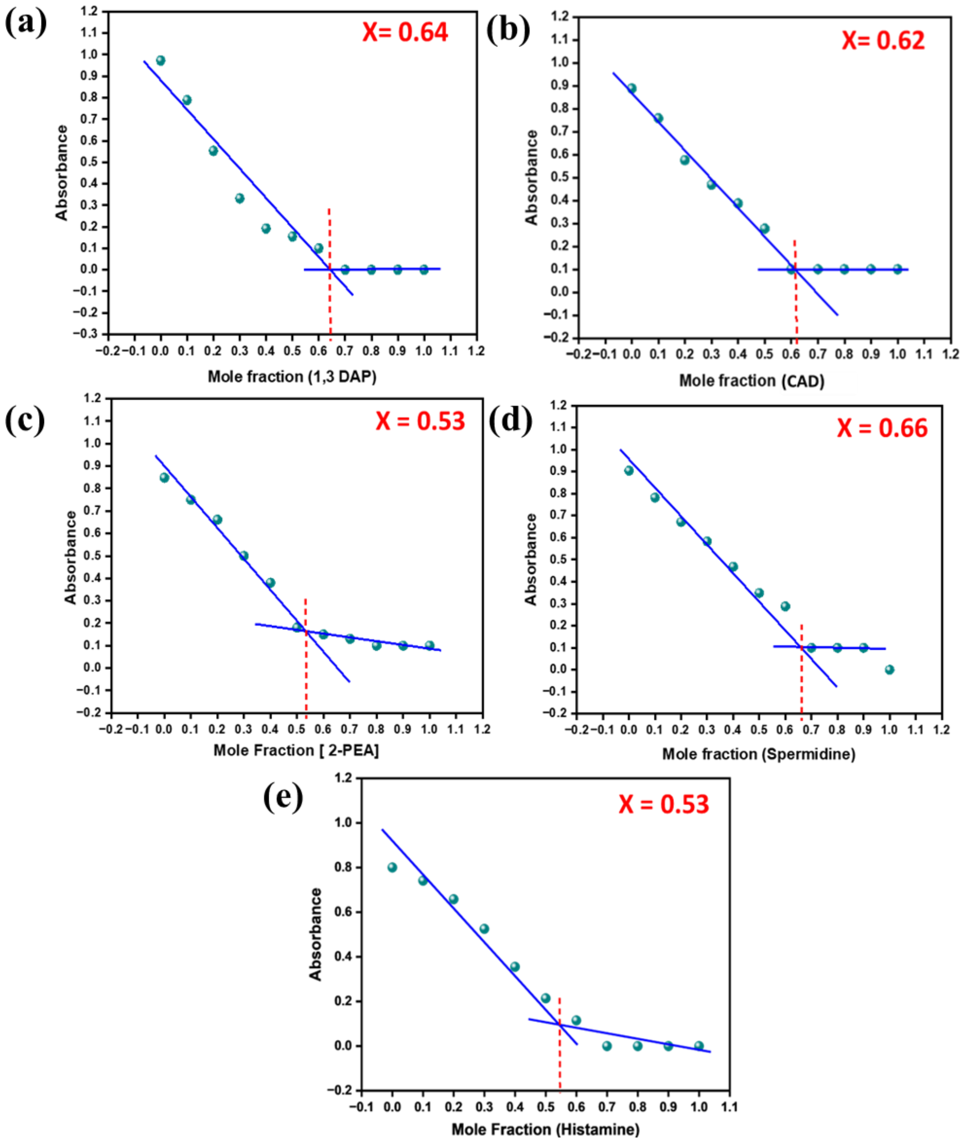

Fig. S11: Job's plot of **PyTPA** with (a) 1,3-DAP, (b) CAD, (c) 2-PEA, (d) spermidine, and (e) histamine depicting stoichiometry with different amines complex with different class of amines.

LC-MS analysis of PyTPA with a variety of amines.

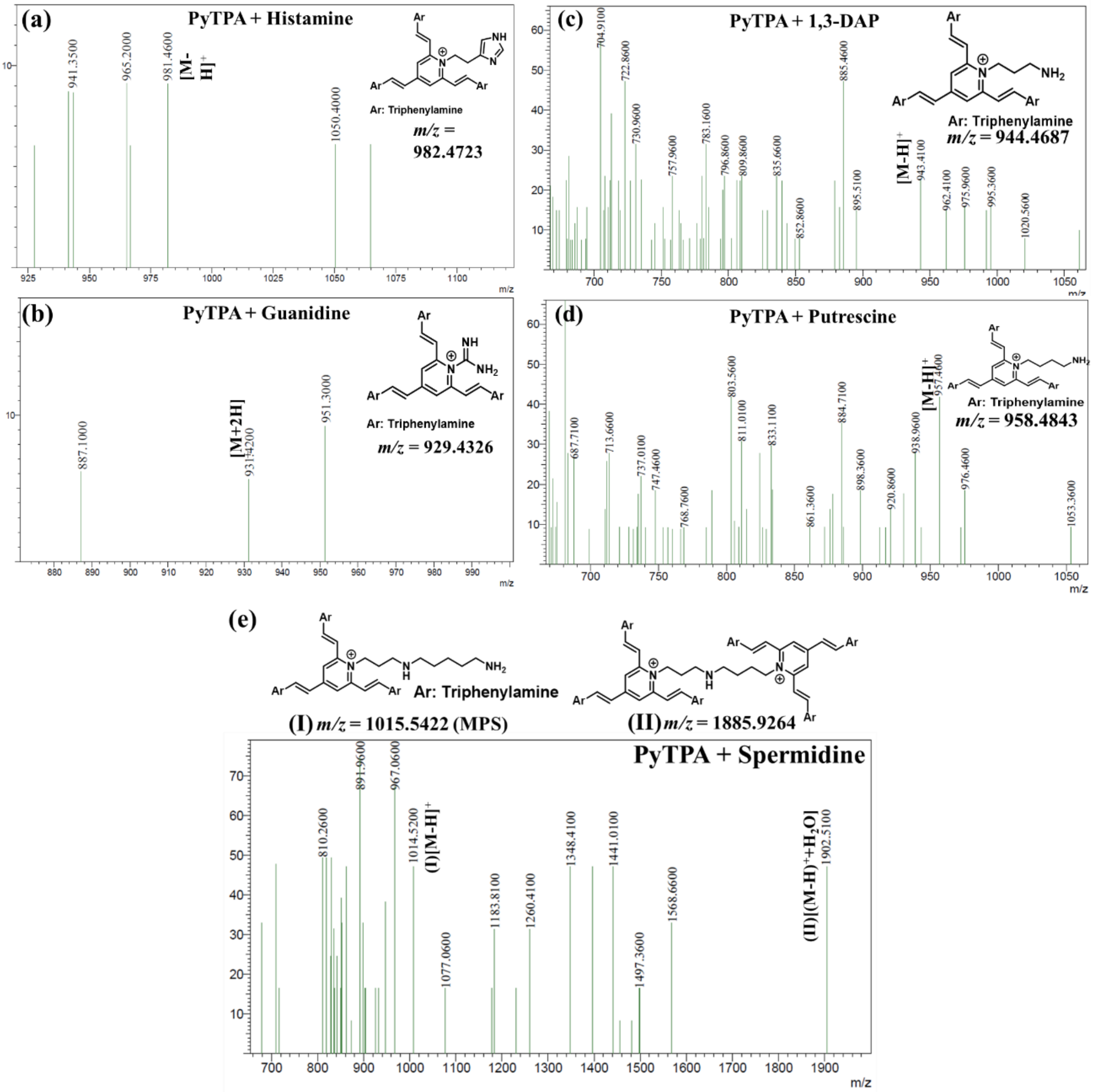

Fig. S12: LC-MS spectra of (a) **PyTPA** + histamine, (b) **PyTPA** + guanidine (c) **PyTPA +** 1,3-DAP (d) **PyTPA** + putrescine. (e) **PyTPA** + spermidine.

^{1}H NMR analysis of PyTPA with Cadaverine

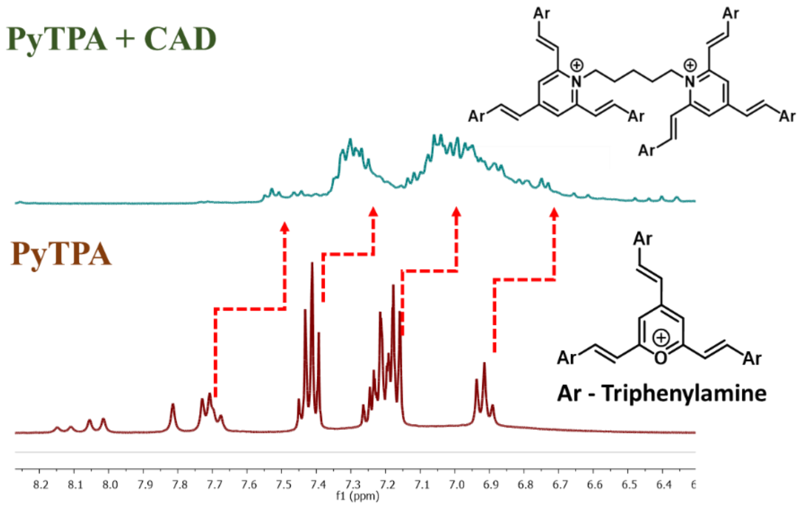

Fig. S13: ^{1}H-NMR (partial) spectra of **PyTPA** in DMSO-d[6] before and after the addition of CAD.

X-ray photoelectron spectroscopy (XPS) study of PyTPA

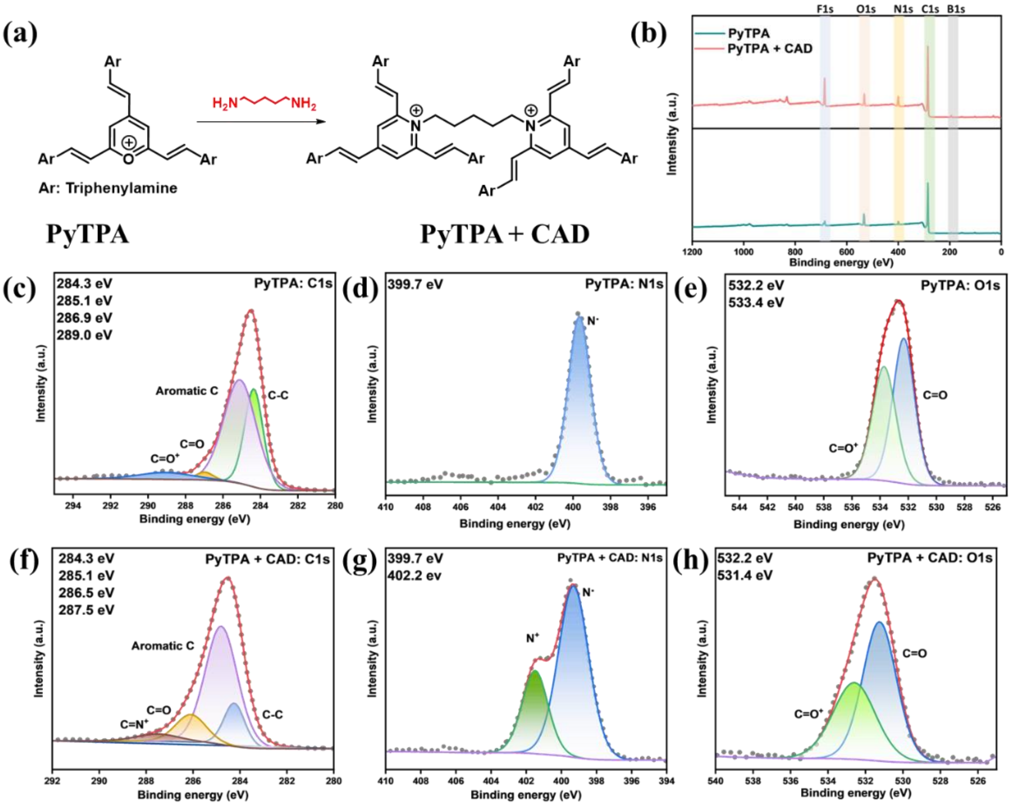

Fig. S14: (a) Schematic representation of reaction of **PyTPA** pyrylium core to form **PyTPA** pyridinium complex with cadaverine, (b) XPS survey spectra for **PyTPA** and complex **PyTPA** + CAD, (c) C1s, (d) N1s, and (e) O1s of **PyTPA,** (f) C1s, (g) N1s, and (h) O1s of **PyTPA +** CAD.

LC-MS spectra of PyTPA with nicotine

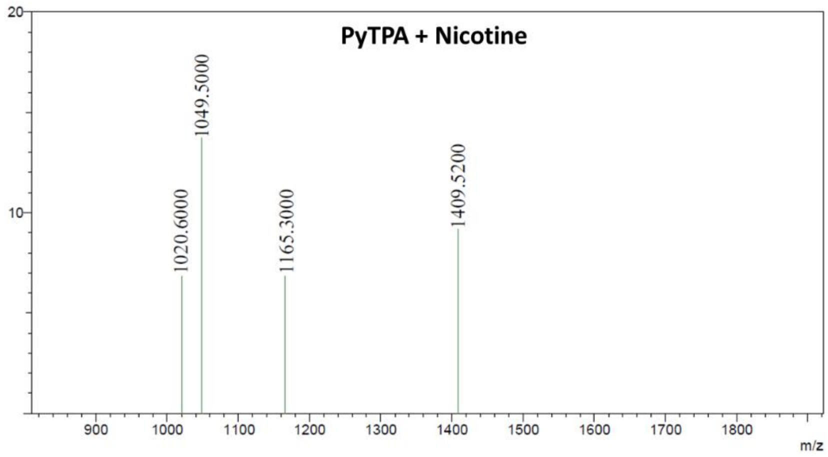

Fig. S15: LC-MS spectra of **PyTPA** with nicotine.

Excited state Lifetime studies

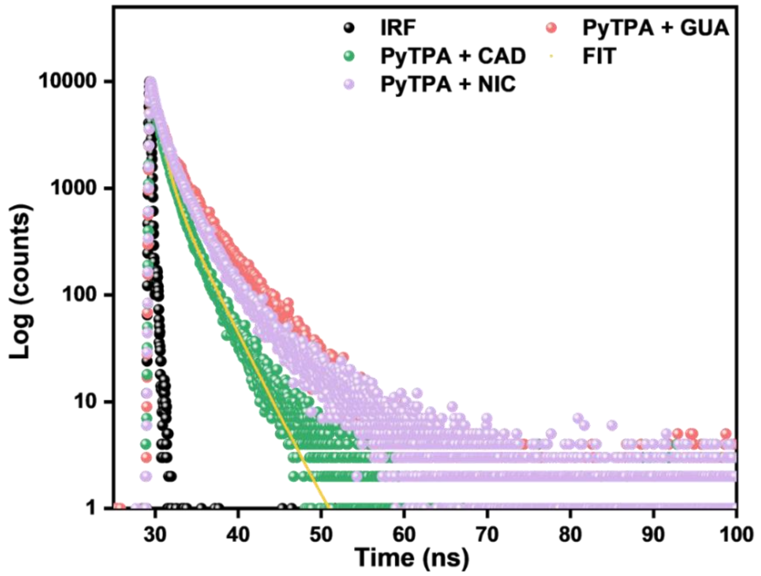

Fig. S16: Lifetime decay plot collected at 530 nm after the addition of amines (10 µM)

Table S3: Lifetime components; All lifetimes (τ) are in ns and the error limit (± 5%)

The average lifetime was obtained by fitting the decay profiles to a bi/tetra-exponential function eq-1.

Fit = $A_1.exp\ (-t/\ \tau_1) + A_2.exp\ (-t/\ \tau_2) + A_3.exp\ (-t/\ \tau_3)$ + …………………..………... (**eq-1**)

A_1, A_2, …. are weighted components, and τ_1, τ_2, and τ_3, …. are individual lifetime components of the decay. The qualities of the fit were determined by judging the chi-square (χ^2) values.

Sample	τ_1	τ_2	τ_3	α_1	α_2	α_3	Avg. <τ>	χ^2
**PyTPA + CAD**	1.52	0.16	4.45	0.22	0.68	0.1	**2.82**	1.1
**PyTPA + GUA**	0.89	2.79	--	0.84	0.16	--	**1.61**	1.1
**PyTPA + NIC**	0.37	1.93	5.95	0.52	0.44	0.04	**2.40**	1.0

Table S4: Lifetime parameters Where, λ_{em} = Emission maxima, Φ_f = quantum yield , τ = Fl. Lifetime, k_r = Radiative rate constant = $\Phi_f/\ \tau$, k_{nr} = Nonradiative rate constant = (1/ τ)- kr.

Sample	λ_{em} (nm)	Φ_f (%)	τ (ns)	k_r (10^9 s^{-1})	k_{nr} (10^9 s^{-1})
**PyTPA + CAD**	530	3.97	2.82	0.014	0.340
**PyTPA + GUA**	530	18.20	1.61	0.113	0.508
**PyTPA + NIC**	530	11.09	2.40	0.046	0.370

The relative quantum yield (Φ_f (%)) of the probe was measured with respect to quinine sulfate (in 0.1 M H_2SO_4) using the formula,

$$\Phi f\ = \Phi ref \times \frac{aref}{asam} \times \frac{Asam}{Aref} \times \left(\frac{nsam}{nref}\right)^2$$

Where, Φ_f = quantum yield of probe Φ_{ref} = quantum yield of quinine sulfate (0.54)

A_{ref} = area under curve of emission spectra of quinine sulfate in 0.1M H_2SO_4, A_{sam} = area under curve of emission spectra of probe, a_{sam} = Absorbance of probe, a_{ref} = Absorbance of quinine sulfate in 0.1M H_2SO_4, n_{sam} = refractive index of DMAc, n_{sam} = refractive index of water.

Table S5: A comparative literature

Sl.No.	Reported work	Detection limit (solution)	Detection limit (vapor) (real-time use-*20mg/L)*	Thermal stability (solid)	Photo stability (soln)	Response time (vapor)	Reference
**1.**	***Multiphase Detection of Crucial Biological Amines using Single 2,4,6-Tristyrylpyrylium Dye***	***37-196 nM***	***4.35 mg/L***	***250 ºC***	***72 hours***	***5 min (Vapor) Instant (Sol)***	***This Work***
2	A Pyrylium Salt-Based Fluorescent Probe for the Highly Sensitive Detection of Methylamine Vapour	84 nM	0.12 mg/L (restricted to only $MeNH_2$)	NA	48 hours	15min (vapor) Instant (Sol)	*Analyst*,2022 **147**, 3451-3455
3	Rapid Visual Detection of Amines by Pyrylium Salts for Food Spoilage Taggant	0.4 µM	5534 mg/L	NA	NA	15 min (vapor) Instant (Sol)	*ACS Appl. Bio Mater*, *2020,* **3**, 772-778.
4	Aggregation Induced Emission Switching Based Ultrasensitive Ratiometric Detection of Biogenic Diamines Using Perylenediimide-Based Smart Fluoroprobe	180-400 nM	28.5 mg/L	NA	NA	60 min (vapour) 120 sec (Sol)	*ACS Appl. Mater. Interfaces,* 2019, **11**, 47207
5	Perylene Bisimide Aggregates as Probes for Sub nanomolar Discrimination of Aromatic Biogenic Amines	~ 10-90 nM	NA	NA	NA	NA	*ACS Appl. Mater. Interfaces,* 2019,**11**, 17079
6	A Highly Sensitive Bimodal Detection of Amine Vapours Based on Aggregation Induced Emission of 1,2- Dihydroquinoxaline Derivatives	40 µM	NA	NA	NA	40 sec (solution)	*Chem. Eur.J.* 2017, **23**, 14911
7	Detection of Amines with Fluorescent Nanotubes: Applications in the Assessment of Meat Spoilage	10-30 nM	NA	NA	NA	60 min (vapor) 30 s (solution)	*ACS Sens.,* 2016, **1**, 22
8	Ylidenemalononitrile Enamines as Fluorescent “Turn-On” Indicators for Primary Amines	25 µM	NA	NA	NA	~ 80 min at room temperatur e (sol)	*J. Am. Chem. Soc.,*2014, **136**, 15493
9	Detection of Biogenic Amines	500 µM	NA	Not mentioned	NA	~ 120 min at 50 ºC (sol)	*Chem. Commun.,* 2011, **47**,

							9639
10	Chromogenic Sensing ofBiogenic Amines	20 µM	NA	NA	24 h	20 min at 60 ºC	*Anal. Chem.*, 2010, **82**, 8402

Note: NA-Not available

Characterisation of PyTPA via HRMS, ^{1}H NMR, ^{13}C NMR and IR.

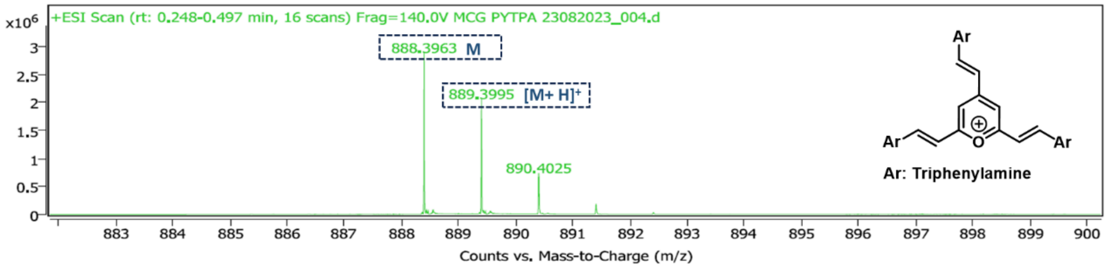

Fig. S17. HRMS spectrum for **PyTPA**

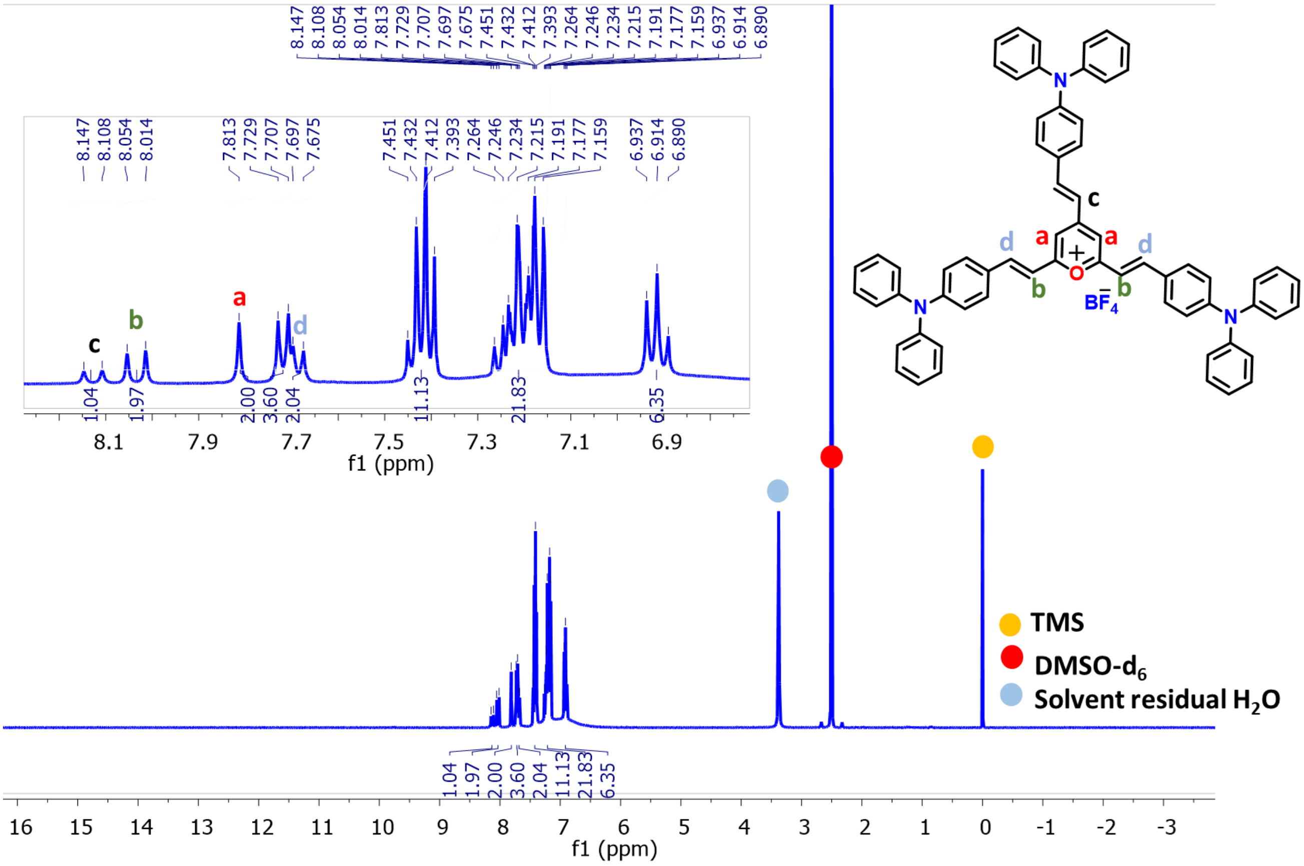

Fig. S18. ^{1}H NMR spectrum of **PyTPA** in DMSO-d_6.

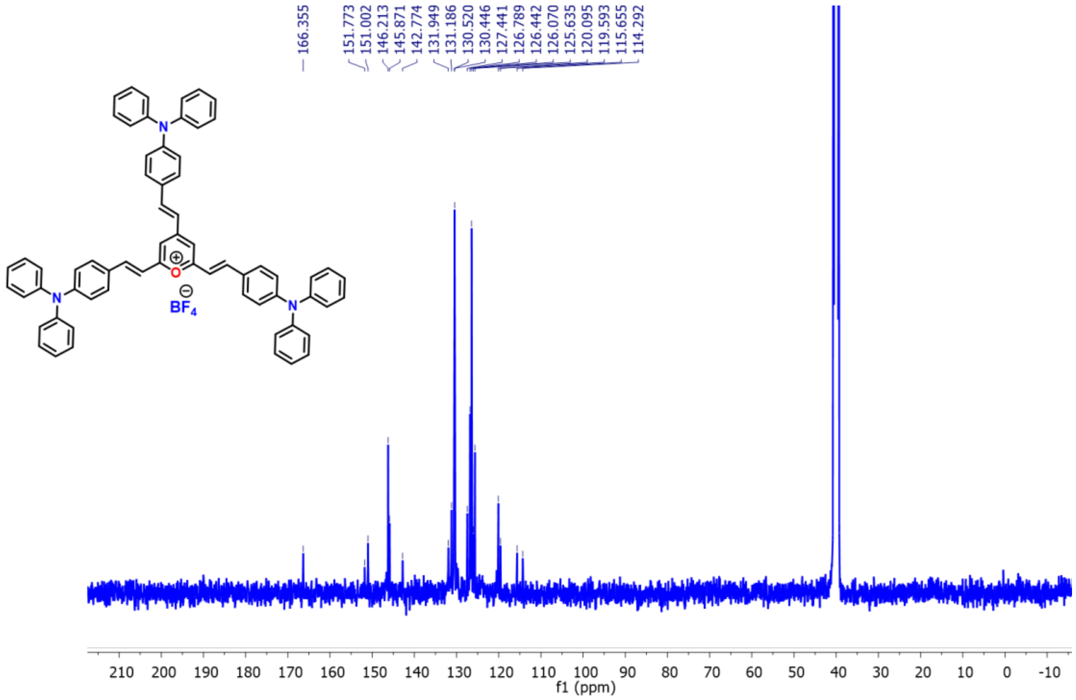

Fig. S19. ^{13}C NMR for **PyTPA** in DMSO-d_6

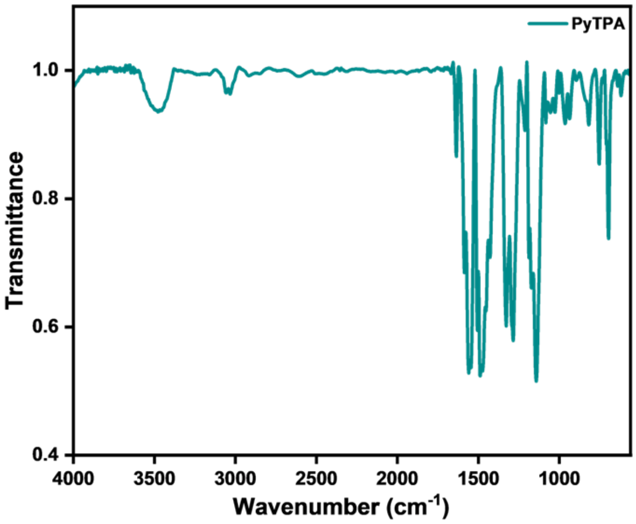

Fig. S20. IR spectrum of **PyTPA.**

____**END**____
