# Supplementary material for: Multiphase detection of crucial biological amines using a 2,4,6-tristyrylpyrylium dye
Source: Commun Chem. 2025 Mar 15;8:81. doi: 10.1038/s42004-025-01459-5 (PMC11910641; doi:10.1038/s42004-025-01459-5)
Supplement: Supplementary file 3 — Description of Additional Supplementary Files [file 42004_2025_1459_MOESM3_ESM.pdf]

Description of Additional Supplementary Files

File name: Supplementary Data 1

Description: All absorption and emission spectra original files
